# Prescription Department

**Published:** 1892-09

**Authors:** 


CHLORAL IN THE TREATMENT OF BOILS.—*Bull. Gen. de Therapeutique.* By *M. Sphen.*

The author recommends very highly as far superior to all other treatment, the use of chloral externally, in this troublesome class of affections. He directs that the boil be kept covered with a tampon of cotton-wool, soaked in the following solution:

℞. Chloral hydrat, oz. iiss.
Aquæ,
Glycerin, aa. f. dr. v.
M.—*American Medical Magazine.*

EMMENAGOGUE.—

An excellent emmenagogue is given in the *Medical News*, Cincinnati, as follows:

℞. Hydrargyra bichloride,
Iodii arsenitis, aa gr. iii.
Strychiniæ sulphatus, gr. ss. (½)
Potassii carbonatis,
Ferri sulphatis, aa gr. xlv.
M. In pilulas lx. divide. S. One pill after each meal.

TAPE WORM.—

*Dr. T. W. Shaw*, of Pittsburg, advises:

℞. Kamala, 1 dr.
Ethereal ext. male fern, 2 dr.
Syr. acaciæ, 1 oz.
M. S. To be given in two doses, one-half on rising in the morning, and the other half two hours afterwards. A dose of castor oil to be given one-half hour after the last dose.—*Ex.*

CHOREA.—

The following was given with great benefit in *DaCosta's Clinic* to a girl suffering from chorea.

℞. Tr. belladonnæ, dr. j.
Liquoris potassii arsenitis, dr. ij.
Syr. hypophosphitis comp. q.s., oz. iij.
S. Teaspoonful ter in die.—From *Medical News.*

FISSURED NIPPLES.—

℞. Balsam Peru, ½ dr.

Tinct. arnica, ½ dr.

Oil sweet almonds, 1 oz.

Lime water, ½ oz.

M. S. Apply a small quantity several times daily.

DIARRHŒA.—

A favorite and old-time prescription for diarrhœa is the following:

R. Tinct. opii, 2 dr.
Tinct. capsici, 2 dr.
Tinct. rhei, 2 dr.
Ess. menth pip., 2 dr.
Spts. camph., 2 dr.

M. S. Fifteen to thirty drops in water; repeated, if necessary, in fifteen to twenty minutes.

HYSTERIA.—

For hysteria in a young woman, aged 18 years, *Prof. Da Costa* prescribed:

R. Zinci valerianat., 2 gr.
Ferri valerianat, 1½ gr.
Ext. belladonna, 1-16 gr.

M. S. One pill three times a day. The patient should also be given tonics, have a full meat diet, and take exercise in the open air.

DIABETES MELLITUS.—

R. Sodii salicylat., 3 dr.
Liq. potass. arsenitis, 1 dr.
Glycerinæ, 1 oz.
Aq. cinam. 3 oz.

M. S. Dessertspoonful three times a day.—*Prac. Monthly.*

CYSTITIS IN WOMEN.—

R. Citrate of potassium, oz. ss.
Fl. ext. of triticum repens,
Tinct. of belladonna, each oz. i.
Fl. ext. buchu, oz. ss.
Water add to make four ounces.

S. A teaspoonful in a wineglassful of water three times a day.—*Journal de Medicine de Paris.*

DROPSY, SCARLATINAL.—By *Dr. R. Denner*, Berne, Switzerland.

R. Diuretin, gr. xxij.
Distilled water, oz. iij.
Brandy, gtt. x.
Sugar, gr. xl.

M. S. To be taken in the course of the twenty-four hours in doses of tablespoonful.

UTERINE SUBINVOLUTION.—By *Prof. B. C. Hirst.* Philadelphia, Pa.

R. Strychninæ sulphatis, gr. 1-20.

Quininæ sulphatis, gr. ij.

Extract ergotæ, gr. j.

M. Ft. pil. No. 1. S. At one dose. To be repeated thrice daily.

RIGID PERINÆUM.—

R. Chloriformi,
Ætheris sulphatis, aa oz. ii.
Listerine, oz. i.

S. Apply locally. It acts quickly and well.

